# The new record of drought and warmth in the Amazon in 2023 related to regional and global climatic features

**DOI:** 10.1038/s41598-024-58782-5

**Published:** 2024-04-06

**Authors:** Jhan-Carlo Espinoza, Juan Carlos Jimenez, José Antonio Marengo, Jochen Schongart, Josyane Ronchail, Waldo Lavado-Casimiro, João Vitor M. Ribeiro

**Affiliations:** 1 Institut des Géosciences de l’Environnement, IRD, CNRS, Université Grenoble Alpes, 70 Rue de La Physique, Bat. OSUG- B. Domaine Universitaire, 38400 Saint Martin d’Hères, France; 2 Instituto de Investigación Sobre la Enseñanza de las Matemáticas (IREM PUCP), Pontificia Universidad Católica del Perú, Lima, 15088 Peru; 3 Global Change Unit (GCU) of the Image Processing Laboratory (IPL), Universitat de València Estudi General (UVEG), C/ Catedrático José Beltrán 2, 46980 Paterna, Valencia, Spain; 4 National Centre for Monitoring and Early Warning of Natural Disasters CEMADEN, Estrada Doutor Altino Bondesan, 500 - Distrito de Eugênio de Melo, São José dos Campos, SP CEP:12.247-060 Brazil; 5 Institute of Science and Technology, São Paulo State University, UNESP, São José dos Campos, SP Brazil; 6 Graduate School of International Studies, Korea University, Seoul, South Korea; 7 Department of Environmental Dynamics, National Institute for Amazon Research (INPA), 2936, Av. André Araújo, Manaus, Amazonas 69067375 Brazil; 8 Laboratoire d’Océanographie et du Climat, LOCEAN-IPSL, IRD, CNRS, MNHN, Sorbonne Université, Paris, France; 9 Servicio Nacional de Meteorología e Hidrología (SENAMHI), Lima, Peru

**Keywords:** Climate sciences, Atmospheric science, Hydrology

## Abstract

In 2023 Amazonia experienced both historical drought and warm conditions. On October 26th 2023 the water levels at the port of Manaus reached its lowest record since 1902 (12.70 m). In this region, October monthly maximum and minimum temperature anomalies also surpassed previous record values registered in 2015 (+ 3 °C above the normal considering the 1981–2020 average). Here we show that this historical dry and warm situation in Amazonia is associated with two main atmospheric mechanisms: (i) the November 2022–February 2023 southern anomaly of vertical integrated moisture flux (VIMF), related to VIMF divergence and extreme rainfall deficit over southwestern Amazonia, and (ii) the June–August 2023 downward motion over northern Amazonia related to extreme rainfall deficit and warm conditions over this region. Anomalies of both atmospheric mechanisms reached record values during this event. The first mechanism is significantly correlated to negative sea surface temperature (SST) anomalies in the equatorial Pacific (November–February La Niña events). The second mechanism is significantly correlated to positive SST anomalies in the equatorial Pacific, related to the impacts of June–September El Niño on the Walker Circulation. While previous extreme droughts were linked to El Niño (warmer North Tropical Atlantic SST) during the austral summer (winter and spring), the transition from La Niña 2022–23 to El Niño 2023 appears to be a key climatic driver in this record-breaking dry and warm situation, combined to a widespread anomalous warming over the worldwide ocean.

## Introduction

Amazonia hosts the Earth’s largest tropical forests characterized by a unique biodiversity. The Amazon basin represents the largest hydrological basin on Earth (about 6.87 million km^2^) with 16–18% of the global freshwater discharge to the oceans. It is the largest and most intense terrestrial convective centre in the Earth system, coupled to global atmospheric circulations^1^. Approximately 13% of global precipitation over the continental areas is concentrated in the Amazon basin, which just accounts for 4.6% of the world's land area^1^. Due to climate change and deforestation this biome is moving towards a “tipping point”, especially in those regions affected by large-scale deforestation, forest fragmentation and degradation over the last decades^2^. Particularly in those regions a warming trend^3,4^, the lengthening of the dry season^5–7^, and a decline of carbon sink^8^ are observed.

**Figure 1:**
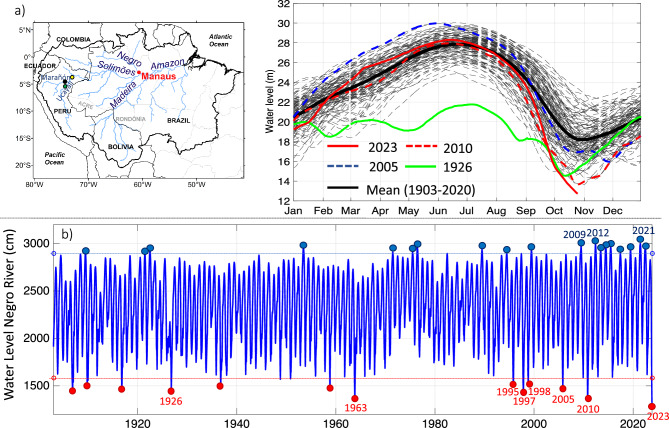
(**a**) Daily water level at the Port of Manaus (Brazil) during the 1903–2023 period (grey dotted lines), mean annual cycle (solid black line) and water level during extreme drought years in 1926 (solid green line), 2005 (dotted blue line), 2010 (dotted red line) and 2023 (solid red line). The black outline on the map indicates the boundary of the Amazonian region. The location of Manaus (red dot), Tamshiyacu (yellow dot), Requena (green dot), San Regis (black dot) and the name of the main rivers, states and countries are indicated in the subpanel. (**b**) Time evolution of the water level of the Rio Negro at Manaus (1903–2023). Years corresponding to extreme drought (flood) with water level below (surpassing) 1580 cm (2900 cm) are indicated with red (blued) dots. All the data come from the platform Hidroweb, available on the National Water Resources Information System (SNIRH) operated by the Brazilian National Water and Sanitation Agency (ANA) and the Geological Survey of Brazil (CPRM). Data visualisations produced using Matlab 2023b (https://matlab.mathworks.com).

At the Port of Manaus (Central Amazonia; Fig. 1a), where daily water level measurements of the Rio Negro exist since September 1902, a state of emergency is declared when the water level is below 15.8 m (a threshold value determined by the Brazilian Geological Survey—CPRM to decree drought). This is due to low water levels that affect navigation and, as a result, the transport of goods and of people living along the riverbanks^9^. On the other hand, extreme floods (when the critical level surpass 29.0 m) were reported with a major frequency and intensity^10,11^, particularly since 2009 (Fig. 1b). However, extreme droughts in Amazonia were also observed in 1997–98, 2005, 2010 and 2015–16^9,12,13^. These historical droughts were associated with El Niño events during the austral summer (December–February) and/or warm conditions in the North Tropical Atlantic during austral winter and spring (June–September)^9,12–14^. These events are related to an increased atmospheric subsidence over Amazonia, associated with anomalies of the Hadley and Walker cells during El Niño^3,15,16^. In addition, a deficit of moisture transport from the Atlantic Ocean toward central and southern Amazonia, and atmospheric subsidence over tropical South America is detected during warm events in the tropical North Atlantic^9,14,17,18^. On the other hand, during the Austral spring and summer, anomalous dry conditions in subtropical South America are related to the weakening of the South American low-level jet east of the Andes modulated by La Niña-related teleconnections^19–21^. Moreover, during multi-years La Niña events (such as the period 2020–22) dry conditions are observed over southwestern Amazonia^21–23^. In particular, a lower frequency of low-level winds is noticed over southwestern Amazonia during La Niña years^19,24^.

Since December 2022, dry situation over the Amazon region coincides with a record-breaking warming, particularly intense since June 2023^25^. The warm and dry conditions in 2023 are causing severe impacts on Amazonian populations and ecosystems^25,26^. Unlike previous extreme droughts, this historical event began during the 2022–23 La Niña event, followed by El Niño during the austral winter and spring of 2023. Therefore, the aim of this study is to document and analyze the regional and large-scale climatic conditions related to this new record of drought and warmth in Amazonia.

## Results

### Main hydrological features of the 2023 drought

Below normal water levels of the Rio Negro at the Port of Manaus were observed in December 2022 and January 2023 (19.10 m; Fig. 1a). During this period southwestern tributaries of the Amazon, such as the Peruvian Ucayali and Amazonas rivers also reported below normal water levels (Fig. S1a,b), reaching the historical December low values in Requena Station in the Ucayali River. In contrast, no anomalies were reported in the northwestern tributaries during the 2022–23 austral summer (e.g., on Marañón River; Fig. S1c). Water levels at the Port of Manaus approach normal values from May to August 2023, during the seasonal flooding period (maximum water level 28.3 mm). However, according to the Brazilian Geological Survey (www.sgb.gov.br) since the end August a rapid decrease was observed, reaching values below the emergency threshold of 15.8 m on September 30th (15.66 m; Fig. 1a). On October 26th the water levels at the port of Manaus reach 12.70 m which is the lowest record since 1902 (Fig. 1b), exceeding the levels recorded during previous historical droughts, such as 13.63 m in 2010, 13.64 m in 1963, 14.37 m in 1997 and 14.75 m in 2005.

This historical drought comes 13 and 18 years after the extreme 2010 and 2005 droughts, respectively^9^. During the last 15 years, the Amazon River has been mainly characterized by extreme floods, such as in 2009, 2012–2015, 2017, 2019 and 2021–2022^11^ (Fig. 1b). The descending water level in 2023 has an amplitude of 15.6 m, the highest on record, which exceeds the long-term average (10.4 m) by 50%. During the last 15 years the fourth highest maximum (2021, 2012, 2009, 2022) and the two lowest minimum (2023, 2010) annual water level have been recorded, concentrated on the last 12.3% of the 122-yr-long long-term instrumental record.

### Regional rainfall patterns during the 2023 extreme drought

**Figure 2:**
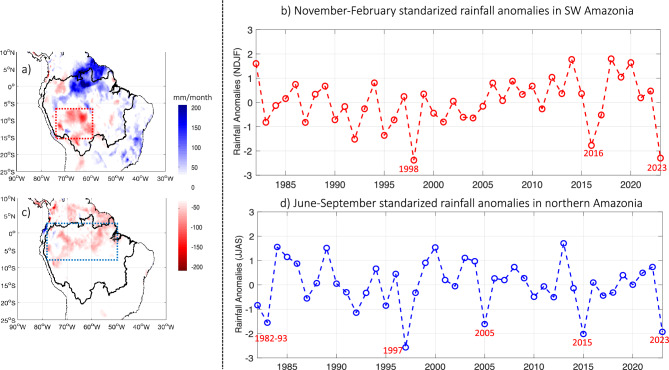
(**a**) Rainfall anomalies for November-February 2022–23 (in mm/month) and (**b**) 1982–2023 standardized rainfall anomalies during the November–February season averaged over the southwestern Amazon Basin (8° S–15° S and 60° W–75° W; dotted red line in panel (**a**). In (**b**) The X axis corresponds to the year of the last month of the November-February season. (**c**) Rainfall anomalies for June–September 2023 (in mm/month) and (**d**) 1982–2023 standardized rainfall anomalies during the June–September season averaged over the Northern Amazon Basin (8° S–2.5° N and 50° W–75° W; dotted blue line in panel (**c**). In (**a**, **c**) anomalies are computed considering the 1982–2020 climatological period and only anomalies higher (lower) than 25 mm/day (− 25 mm/day) are plotted. Data visualisations produced using Matlab 2023b (https://matlab.mathworks.com).

Rainfall patterns during the 2022–23 hydrological year in the Amazon basin were characterized by significant negative anomalies over southwestern Amazonia during the November 2022–February 2023 season (Bolivian and southern Peruvian Amazon and Rondônia and Acre states in Brazilian Amazon; Fig. 2a). This rainfall pattern can be related to the low water level values in Tamshiyacu and Requena stations in the Ucayali and Amazonas rivers in Peru (Fig. S1a,b). During this season the regional average rainfall anomaly over southwestern Amazonia (8° S–15° S and 60° W–75° W; Fig. 2a) reached − 41 mm/month, which is an historical record that was exceeded only during the 1998 El Niño year (− 42 mm/month). Very low rainfall anomalies are also reported during other El Niño years, such as in 2016 and 1992 (Fig. 2b).

In the June–September season of 2023 negative rainfall anomalies prevailed in the northern part of South America, including northern Amazonia (Fig. 2c). This season is the regional dry period in Amazonia^27^. Negative rainfall anomalies in northern Amazonia (8° S–2.5° N and 50° W–75° W; Fig. 2c) reach − 25 mm/month in June–September 2023. This value was only surpassed in 1997 (− 34 mm/month) and is comparable to the anomaly of 2015 (− 26 mm/month) but was even lower than that of 2005 (− 21 mm/month) and 1983 (− 20 mm/month). As can be seen in Fig. 2d, all the above-mentioned years, characterized by significant negative rainfall anomalies over northern Amazonia, correspond to El Niño conditions during the June–September season. In 2023, El Niño conditions were reported since the March–May season, and very warm SST anomalies were observed in the equatorial Pacific during June to September season, reaching + 2.3 °C of SST anomalies in the Niño 3.4 region (5° N–5° S; 120° W–170° W) in November 2023 (Fig. S2).

In summary, two regions were affected by extreme dry conditions in Amazonia during the 2022–23 hydrological year, the southwestern Amazon in November 2022–February 2023, where the lowest rainfall value was reported, and the northern Amazonia in June–September 2023, where the second lowest rainfall value was observed (Fig. 2).

### Record-breaking warming in Amazonia during 2023

**Figure 3:**
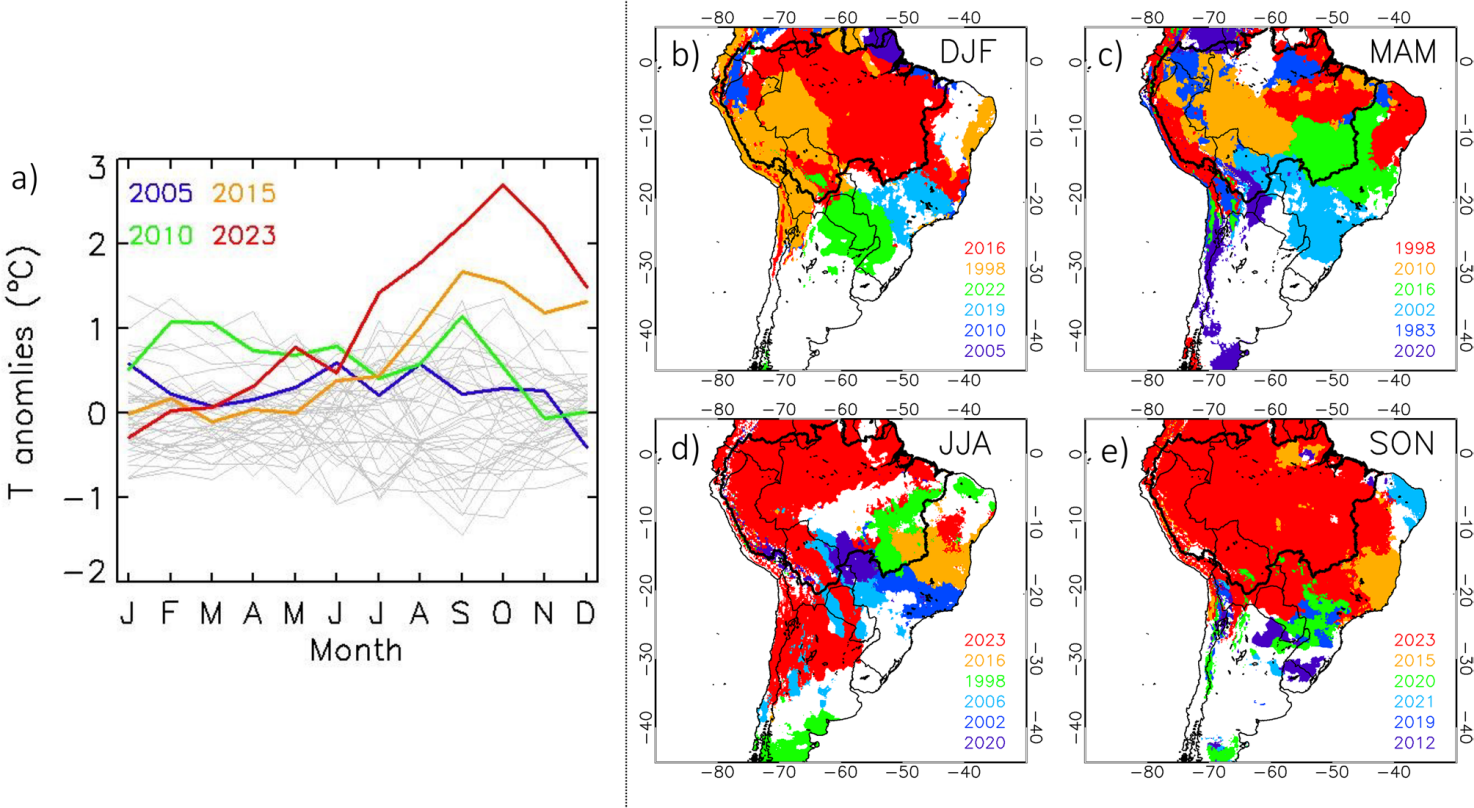
(**a**) Interannual monthly air temperature anomalies for the period 1980–2023 averaged over the Amazon Basin. Monthly time series are provided for each year (grey lines), and for some selected years characterized by high temperature anomalies, including 2023 (2005, blue line; 2010, green line; 2015, orange line; 2023 red line). (**b**–**e**) Maps of regions where maximum seasonal temperatures occurred during the six warmest years during the 1980–2023 period: (**b**) December to January (DJF), (**c**) March to May (MAM), (**d**) June to August (JJA), and (**e**) September to November (SON) seasons. The thick black line delineates the Amazon Basin. Data visualisations produced using IDL v8 (https://www.nv5geospatialsoftware.com/Products/IDL).

The Amazon Basin experienced record values of temperatures during 2023. August, September and October provided the highest air temperature anomaly since 1980, with values of + 1.8, + 2.2, and 2.7 °C, respectively, thus surpassing previous record values (Fig. 3a). These results are corroborated with air temperature measurements at the meteorological station of Manaus, with October providing the highest anomaly (~ + 3 °C both for maximum and minimum air temperatures) since 1960 (Fig. S3). For the current period from January to October, average air temperature anomalies in 2023 (+ 0.95 °C) also surpassed previous record values observed in 2010 (0.78 °C) and, therefore values obtained during other previous extreme drought events (Fig. 3a). When we analyze the spatial distribution of the last year a record temperature was broken (Fig. 3b–e), we observe that 2023 was the hottest year since at least 1980 in JJA and SON seasons over most parts of Amazonia. More specifically, for JJA season this warming is focused on northern and western Amazonia (Fig. 3d), but in SON season this warming extended to almost all the region (Fig. 3e). It is worth mentioning that during the DJF (Fig. 3b) and MAM seasons (Fig. 3c), other warm events (e.g., El Niño 1998 at DJF and MAM, El Niño 2016 at DJF and the 2010 drought at MAM) provided higher temperature anomaly values than those observed in 2023, so the spatial pattern was more heterogeneous during the wet or wet to dry transitions seasons than during the dry seasons.

### Main features of the ocean-atmospheric patterns during the extreme warm and dry 2023

**Figure 4:**
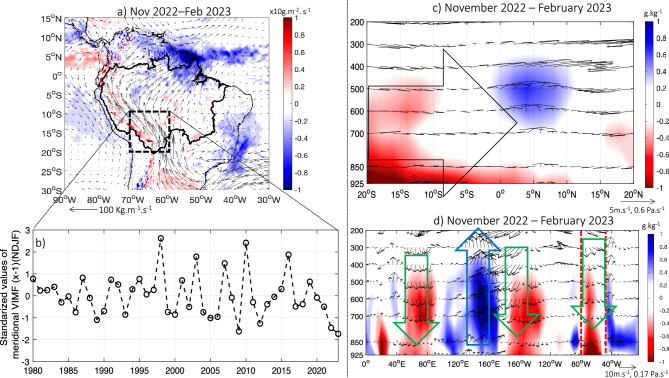
(**a**) Anomalies of vertically integrated water vapor flux (vectors) and its divergence (colors) during November 2022 to February 2023. Only anomalies of divergence of vertically integrated water vapor flux higher (lower) than 0.1 are plotted. (**b**) November (n − 1)—February (n) temporal evolution of the meridional component of the vertical integrated moisture flux (VIMF; standardized values multiplied by − 1 or VIMFx-1) in the region 10° S–20° S and 71° W–60° W (black box in panel (**a**) for the period 1980–2023 period. (**c**) November 2022–February 2023 anomalies of specific humidity (colors) and meridional-vertical wind (vector) in a latitude–pressure level cross section averaged between 75 and 50° W. (**d**) November 2022–February 2023 anomalies of the specific humidity (colors) and zonal-vertical wind (vector) in a longitude–pressure level cross section between 10 and 20° S. Anomalies are computed considering the 1982–2020 climatological period. Black arrow in (**b**) outlines zones with predominance of southern wind anomalies. Green (blue) arrow in (**c**, **d**) outlines zones characterized by atmospheric downward motion (upward motion) anomalies. Only anomalies of the specific humidity higher (lower) than 0.2 g/kg (− 0.2 g/kg) are plotted. Red vertical lines in (**d**) indicate the boundaries of the Amazon basin (75° W–50° W). Data visualisations produced using Matlab 2023b (https://matlab.mathworks.com).

November 2022–February 2023 season is characterized by an exceptional anomalous southerly vertically integrated moisture flux (VIMF) over the Amazonia, particularly intense over its southwestern part (Fig. 4a). This atmospheric feature corresponds to a deficit of moisture from the central and northern Amazonia, which is a key mechanism related to rainfall scarcity over southwestern Amazonia^6,21–24,28^. This moisture deficit reaches record values in November 2022—February 2023 (Fig. 4b).

Analyzing a latitude-pressure level cross section, Fig. 4c shows negative anomalies of specific humidity over southern Amazonia (20° S–10° S) associated with the above-described southerly anomalies of VIMF. In addition, a longitude-pressure level cross section shows and intensification of downward motion over southwestern Amazonia during November 2022–February 2023 (Fig. 4d), while upward motion and convergence of VIMF is observed in the Equatorial Atlantic (Fig. 4a,c). These atmospheric features, characterized by an exceptional deficit of VIMF and downward motion over southwestern Amazonia are related to the remarkable rainfall deficit over southwestern Amazonia during November 2022–February 2023, related to the La Niña event at that time (Figs. 2a, 3b).

**Figure 5:**
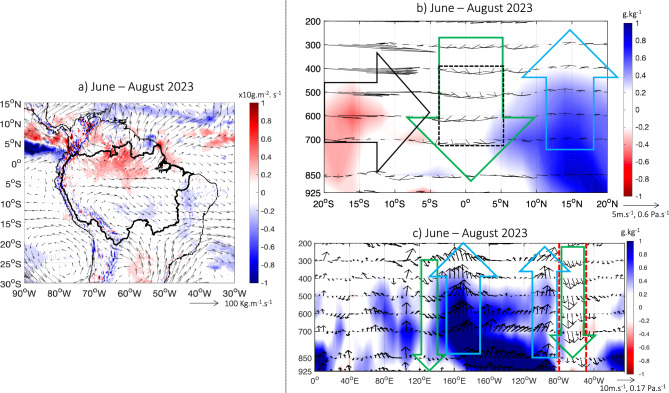
(**a**) Anomalies of vertically integrated water vapor flux (vectors) and its divergence (colors) during June–August 2023. Only anomalies of divergence of vertically integrated water vapor flux higher (lower) than 0.1 are plotted. (**b**) June–August 2023 anomalies of specific humidity (colors) and meridional-vertical wind (vector) in a latitude–pressure level cross section averaged between 75 and 50° W. Black box on b) outline the region where standardized values of Omega are averaged and presented in Fig. 6e. (**c**) June–August 2023 anomalies of the specific humidity (colors) and zonal-vertical wind (vector) in a longitude–pressure level cross section between 5° N and 5° S. Anomalies are computed considering the 1982–2020 climatological period. In (**b**, **c**) black arrow outlines zones with predominance of southern wind anomalies, while green (blue) arrow outlines zones characterized by atmospheric downward motion (upward motion) anomalies. Only anomalies of the specific humidity higher (lower) than 0.2 g/kg (− 0.2 g/kg) are plotted. Red vertical lines in c) indicate the boundaries of the Amazon basin (75° W–50° W). Data visualisations produced using Matlab 2023b (https://matlab.mathworks.com).

During June–August 2023 southern anomaly of VIMF are observed in the North of the continent and over the north Tropical Atlantic Ocean (Fig. 5a). This atmospheric pattern reduces the moisture transport from the Atlantic Ocean to the northern and central Amazonia, which is generally associated with a decrease (increase) convective activity in the central and northern Amazonia (North Tropical Atlantic)^14^. Indeed, over northern Amazonia, divergence of VIMF is particularly intense during this season (Fig. 5a). The latitude-pressure level cross section analysis (Fig. 5b) revels a downward (upward) intensification over northern Amazonia (north Tropical Atlantic), which can be interpreted as an intensification of the continental Hadley circulation^14^. In addition, southern VIMF anomalies remain over southwester Amazonia. These atmospheric features are consistent with negative rainfall anomalies and warm conditions over northern Amazonia during June–September season of 2023 (Figs. 2b, 3d). Two heat waves accentuated the warm conditions; they affected central South America, including southwest Amazonia in August and September 2023 as it is explained in technical reports from some federal government agencies working on climate and hydrology (INMET-www.inmet.gov.br; INPE-www.inpe.gov.br and CEMADEN-www.cemaden.gov.br). In Bolivia, average maximum temperatures during July–September 2023 were 0.5 to 3.5 °C above normal; these values were particularly high in September, with variations ranging from + 3 °C in La Paz to + 7 °C in the Chaco (www.senamhi.gob.bo).

**Figure 6:**
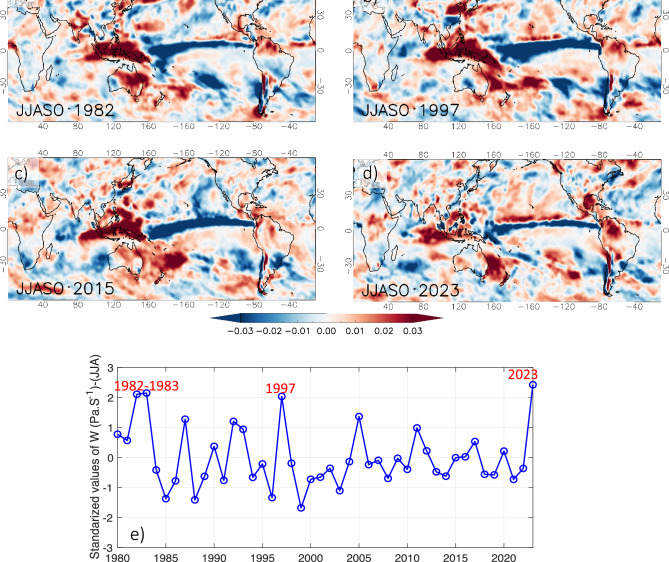
Anomalies of vertical velocity w (Pa/s) at 500 hPa for the season June to October (JJASO) during the four major El Niños: (**a**) 1982, (**b**) 1997, (**c**) 2015 and (**d**) 2023. (**e**) 1980-2023 standardized values of vertical velocity averaged over the northern Amazonia (5° N–5° S; 50° W–75° W; dotted black box on Fig. 5b). Data visualisations for panels (**a**–**d**) produced using IDL v8 (https://www.nv5geospatialsoftware.com/Products/IDL). Data visualisations for panel (**e**) produced using Matlab 2023b (https://matlab.mathworks.com).

Analyzing a longitude-pressure level cross section, Fig. 5c shows an intensification of downward (upward) motion over northern Amazonia (equatorial Pacific) during June–August 2023, which is a usual anomaly of the Walker Circulation observed during El Niño years^18,29^. Compared with previous dry years in northern Amazonia during the June–September season (Fig. 2d), Fig. 6a–d shows a common large-scale pattern with downward (upward) motion at 500 hPa over northern Amazonia and Indo-Pacific region (equatorial Pacific). In northern Amazonia, the downward motion was particularly intense in 1982–83, 1997 and reached a record in 2023 (Fig. 6e).

In summary, two atmospheric mechanisms were exceptional in Amazonia during the 2022–23 hydrological year, on the one hand the November 2022–February 2023 southern anomaly of VIMF (Fig. 4b), related to divergence of VIMF and the extreme rainfall deficit over southwestern Amazonia, and on the other hand, the June–August downward motion over northern Amazonia (Fig. 6e) related to extreme rainfall deficit and warm conditions over Amazonia. The possible links between these atmospheric features and global SST are investigated in the next section.

### Sea surface temperatures related to the main atmospheric features during the extreme warm and dry 2023

**Figure 7:**
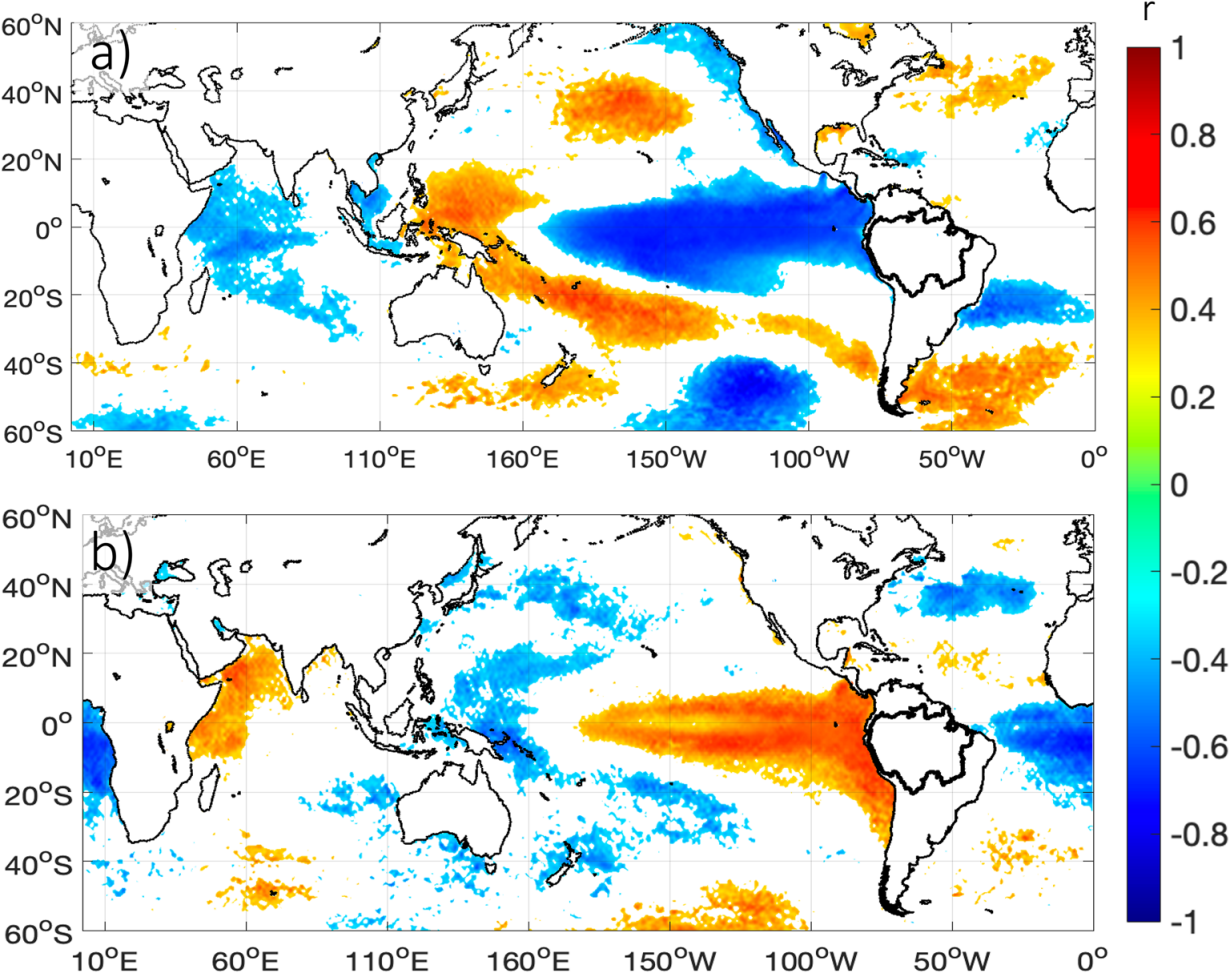
1980–2023 correlation coefficient between: (**a**) November (n − 1) to February (n) meridional component of the vertically integrated moisture flux averaged in the region 10° S–20° S and 71° W–60° W (black box in Fig. 4a; temporal series shown in Fig. 4b) versus November (n – 1) to February (n) SST. (**b**) June to August vertical velocity averaged over the northern Amazonia (5° N–5° S; 50° W–75° W; dotted black box on Fig. 5b and temporal series shown in Fig. 6e) versus June to August SST. Only significant correlation values at *p* < 0.05 are plotted. The continents and boundaries of Amazonia are shown in black line. Data visualisations produced using Matlab 2023b (https://matlab.mathworks.com).

Southerly VIMF anomalies in southwestern Amazonia during November–February season (Fig. 4b) are significantly and negatively (positively) correlated with the central (western) equatorial Pacific SST. This result indicates that during La Niña (El Niño) events southerly (northerly) VIMF anomalies characterize this region at the east of the Andes (Fig. 7a). The correlation maps of Fig. 7a shows that this correlation is higher over the central equatorial Pacific (100° W–180° W). This result is consistent with the negative (positive) anomalies of VIMF shown in Fig. 4b in 1984, 1999, 2009 and 2012 (1982–83, 1998, 2010 and 2016), all of them showing cold (warm) conditions in the central equatorial Pacific, with a record value during La Niña 2022–2023.

Downward motion over northern Amazonia during the June–August season (time series in Fig. 6c) appears significantly and positively (negatively) correlated with the eastern tropical and central equatorial Pacific SST (western equatorial Pacific and south Tropical Atlantic SST) (Fig. 7b). This result indicates that downward (upward) motion over northern Amazonia occurs during El Niño (La Niña) conditions, which is part of the anomalies of the Walker circulation during El Niño (La Niña)^7,18,29^. This result agrees with Fig. 6e, where the highest values of downward motion are observed in 1982–1983, 1997 and 2023, all of them characterized by warm conditions concentrated in the eastern and central equatorial Pacific.

## Conclusions and final comments

Two climatic records were reported in Amazonia in 2023, including the lowest water level in October in the Port of Manaus since 1902 and the warmest air temperatures since at least the last 50 years during the June–October season. These events are taking place at a time when the Amazon rainforest is moving towards a “tipping point”, especially those regions undergoing intense forest degradation and deforestation, characterized by a warming trend^3,4^, a lengthening of the dry season^5,7^ and a decline of the Amazon carbon sink^1,8^. Both, climate change and Amazon deforestation, are considered as main drivers of this biophysical transition in Amazonia^20–36^.

The historical dry and warm situation in Amazonia in 2023 is associated with two main atmospheric situations: on the one hand, a strong southern anomaly of vertical integrated moisture flux (VIMF) over southwestern Amazonia during the November 2022–February 2023 season. This specific regional atmospheric circulation produces a deficit of humidity, VIMF divergence and, consequently, extreme dry conditions over southwestern Amazonia. In contrast to previous extreme summertime dry conditions over this region (observed during El Niño years of 1998 and 2016), this event occurred during a mature phase of the 2022–23 La Niña event. Our results show that this mechanism associated with dry conditions in southwestern Amazonia (i.e., southern anomaly of VIMF) is significantly correlated to negative SST anomalies in the equatorial Pacific (i.e., La Niña events), as observed in 2022–23. This atmospheric feature has been observed during previous La Niña events, for instance in 1999, 2009 and 2012. In agreement, studies have reported a deficit of precipitation during the September–November season over the South American Altiplano and southwestern Amazonia during La Niña years^21–23,37,38^, associated with southerly anomalies and divergence of VIMF^19–22^.

On the other hand, this study describes a record in atmospheric downward motion over northern Amazonia during June–August 2023. This atmospheric situation is associated with atmospheric stability, extreme rainfall deficit, and warm conditions over this region. This atmospheric feature has been observed in previous warm and dry years in northern Amazonia, such as 1982–83; 1997, 2005, reaching record values in 2023. Atmospheric downward motion over northern Amazonia is significantly and positively correlated with the eastern tropical and central equatorial Pacific SST. Indeed, previous studies have documented the relationship between El Niño (La Niña) and atmospheric downward (upward) motion over northern Amazonia, as part of the impacts of El Niño southern Oscillation on Walker Circulation^10,18–20^. These results suggest that the transition from La Niña during November 2022–February 2023 (related to negative rainfall anomalies in southwestern Amazonia) to El Niño 2023 (related to negative rainfall anomalies in northern Amazonia) acted as the main climatic drivers in this historical 2023 dry and warm situation in Amazonia.

While the above documented atmospheric features (i.e., southerly VIMF anomalies over southwestern Amazonia and downward motion in northern Amazonia) are related to SST variability, the fact that in 2023 we observed record values of both mechanisms is not fully explained by SST variability. Indeed, dry and warm conditions observed during some extreme El Niño years (1982–83; 1998, 2015–16) were surpassed in 2023. Therefore, further analysis is required to examine the influence of other global-scale processes (e.g., global warming^31,32,39,40^) or regional-scale factors, such as Amazon deforestation, in relation to this extreme dry and warm event. High deforestation rates regionally affect the hydrological cycle in the Amazon basin^41–43^. Deforestation reduces the latent heat (evapotranspiration) and increases the sensible heat, contributing to the heating of the lower atmosphere and reducing the amount of water vapor in the atmosphere^33–36^. In addition, the hot and dry conditions favor the occurrence of anthropogenic large-scale fires^7,44^. In consequence of the reduced water recycling by the forest vegetation^45,46^ and the synergies and feedback in the land–atmosphere coupled system, deforestation induced hydroclimatic drought covering almost the entire Amazon basin^29,46–48^. This question deserves further analysis associated with the attribution of global warming and Amazon deforestation to warmth and dry extremes in Amazonia.

## Methods

### Water level records

Daily water level records of the Rio Negro for the Port of Manaus since September 1902 come from the Hidroweb platform of the National Water Resources Information System (SNIRH) operated by the Brazilian National Water Agency (ANA) and the Geological Survey of Brazil (CPRM) and daily actualized on the platform https://www.portodemanaus.com.br/?pagina=nivel-do-rio-negro-hoje. Note that water levels at Manaus station are controlled by the Rio Solimões water level variability due to the backwater effect^49^.

We identify in the daily water level time-series of the Port of Manaus values above and below the thresholds of 29.0 m and 15.8 m, which correspond to the emergency levels for floods and droughts respectively^50^. In addition, daily water level records in three Peruvian hydrological stations were obtained from the Peruvian National Meteorological and Hydrological Institute (SENAMHI), for three Peruvian hydrological stations: Tamshiyacu (Amazonas River; since 1984), San Regis (Marañón River, since 1986) and Requena (Ucayali River, since 1995).

### Precipitation data

We use precipitation dataset from the Climate Hazards Group Infrared Precipitation with Stations (CHIRPS)^51^. This rainfall dataset, with information available since 1981, combines satellite and rain gauges data and uses the global cold cloud duration (CCD) as a thermal infrared method to estimate the global precipitation. Gridded rainfall time series at a high horizontal resolution (0.05°) is obtained incorporating to CHIRPS climatology (CHPclim) satellite imagery, and in-situ station data. According to scientific literature, CHIRPS is a suitable satellite precipitation product to analyze rainfall variability over the Amazon Basin^52–54^. Here we use the CHIRPS V2.0 dataset at monthly time step, which is available at: https://data.chc.ucsb.edu/products/CHIRPS-2.0/global_daily/.

### Ocean–atmosphere data

Ocean and atmosphere data were extracted from the European Centre for Medium-Range Weather Forecasts ECMWF Reanalysis Version 5 (ERA5)^55^. ERA5 reanalysis provides different land/sea and atmospheric fields at hourly time-steps and spatial grids of 0.25º from 1950 to present, although our study period focusses on monthly values over the period 1980–2023. More specifically, we used zonal and meridional wind, vertical velocity (omega component), and specific humidity at different pressure levels (1000–200 hPa). Values of vertically integrated water vapor flux (VIMF) and its divergence were also included in our analysis. Ocean conditions were assessed through monthly Sea Surface Tempearture (SST) anomalies, and air temperature at 2 m was obtained from the land component of the ERA5 reanalysis (ERA5-Land). ERA5-Land includes a series of improvements including an enhanced grid resolution of 1º × 1º making it more accurate for all types of land applications^56^. Data used in this study is available from the Climate Data Store (CDS) of the Copernicus Climate Change Service (C3S) (https://cds.climate.copernicus.eu/). These temperature fields are generated at a spatial grid of 0.25º and are available from 1950 to the present.

### Statistical methods

In this study, monthly anomalies of rainfall and atmospheric variables are computed considering the period 1982–2020 as a climatological average, whereas SST and air temperature anomalies are computed using the period 1981–2020 as a climatological average. Time series of monthly values are used to analyze interannual variations of different parameters, and seasonal values are also used to analyze the spatial patterns across the Amazon Basin. Typical climatological seasons (three monthly) DJF, MAM, JJA and SON were considered, as well as some specific seasonal averages such as NDJF and JJASO. Statistical relationships between SST and atmospheric variables are computed using linear Pearson correlation coefficient (r) for the period 1982–2023. Throughout the study statistical methods were carried out using Matlab 2023b.

### Supplementary Information

Supplementary Information.

## Data Availability

The ocean–atmosphere datasets analyzed during the current study are available in the Copernicus Climate Change Service repository (https://cds.climate.copernicus.eu/). Precipitation dataset analyzed during the current study is available in the Climate Hazards Center (CHC) repository (https://data.chc.ucsb.edu/products/CHIRPS-2.0/global_daily/). Water level records of the Rio Negro analyzed during the current study are available in the Hidroweb repository of the National Water Resources Information System (SNIRH) by the Brazilian National Water Agency (ANA) and the Geological Survey of Brazil (CPRM) (https://www.portodemanaus.com.br/?pagina=nivel-do-rio-negro-hoje). Finally, water level records of Peruvian Rivers analyzed during the current study are available in SENAMHI-Peru repository (https://www.senamhi.gob.pe/?p=estaciones).
