# Supplementary material for: The new record of drought and warmth in the Amazon in 2023 related to regional and global climatic features
Source: Sci Rep. 2024 Apr 6;14:8107. doi: 10.1038/s41598-024-58782-5 (PMC10998876; doi:10.1038/s41598-024-58782-5)
Supplement: Supplementary file 1 — Supplementary Information. [file 41598_2024_58782_MOESM1_ESM.docx]

**The new record of drought and warmth in the Amazon in 2023 related to regional and global climatic features**

Jhan-Carlo Espinoza^1,2*^, Juan Carlos Jimenez^3^ José Antonio Marengo^4,5,6^, Jochen Schongart^7^, Josyane Ronchail^8^ Waldo Lavado^9^, João Vitor M. Ribeiro^5^

1.- Institut des Géosciences de l’Environnement, Université Grenoble Alpes, IRD, CNRS.

70 Rue de la Physique, Bat. OSUG- B. Domaine Universitaire 38400 Saint Martin d'Hères, France.

2.- Instituto de Investigación sobre la Enseñanza de las Matemáticas (IREM PUCP). Pontificia Universidad Católica del Perú, Lima, 15088, Peru.

3.- Global Change Unit (GCU) of the Image Processing Laboratory (IPL), Universitat de València Estudi General (UVEG), C/ Catedrático José Beltrán 2, 46980 Paterna, Valencia, Spain.

4.- National Centre for Monitoring and Early Warning of Natural Disasters CEMADEN, Estrada Doutor Altino Bondesan, 500 - Distrito de Eugênio de Melo, São José dos Campos/SP, CEP:12.247-060. Brazil.

5.- Institute of Science and Technology, São Paulo State University, UNESP. São José dos Campos, SP, Brazil

6- Graduate School of international Studies. Korea University, Seoul, South Korea

7.- National Institute for Amazon Research (INPA), Department of Environmental Dynamics, 2936, Av. André Araújo, Manaus, Amazonas, 69067375. Brazil.

8.- Laboratoire d'Océanographie et du Climat, LOCEAN-IPSL, Sorbonne

Université, IRD, CNRS, MNHN, Paris, France

9.-Servicio Nacional de Meteorología e Hidrología (SENAMHI), Lima. Peru

**Supplementary Information**

**Supplementary Figure 1.** Daily water levels for each year between 2023 and 1984, 1995 and 1986 in a) Tamshiyacu (Amazonas River), b) Requena (Ucayali River) and c) San Regis (Marañón River) stations in Peruvian Amazon, respectively. The red line represents the water level during the 2022-23 hydrological year. Missing values during 2022-23 are due to missing observations of water level in these hydrological stations.

**Supplementary Figure 2.** Seasonal sea surface temperature anomalies in 2022 and 2023. From West to East, the black boxes refer to the western and eastern equatorial Indian Ocean regions, the Niño 3.4 region, and the tropical North Atlantic region. Grey boxes delineate Niño 4 and 3 regions.


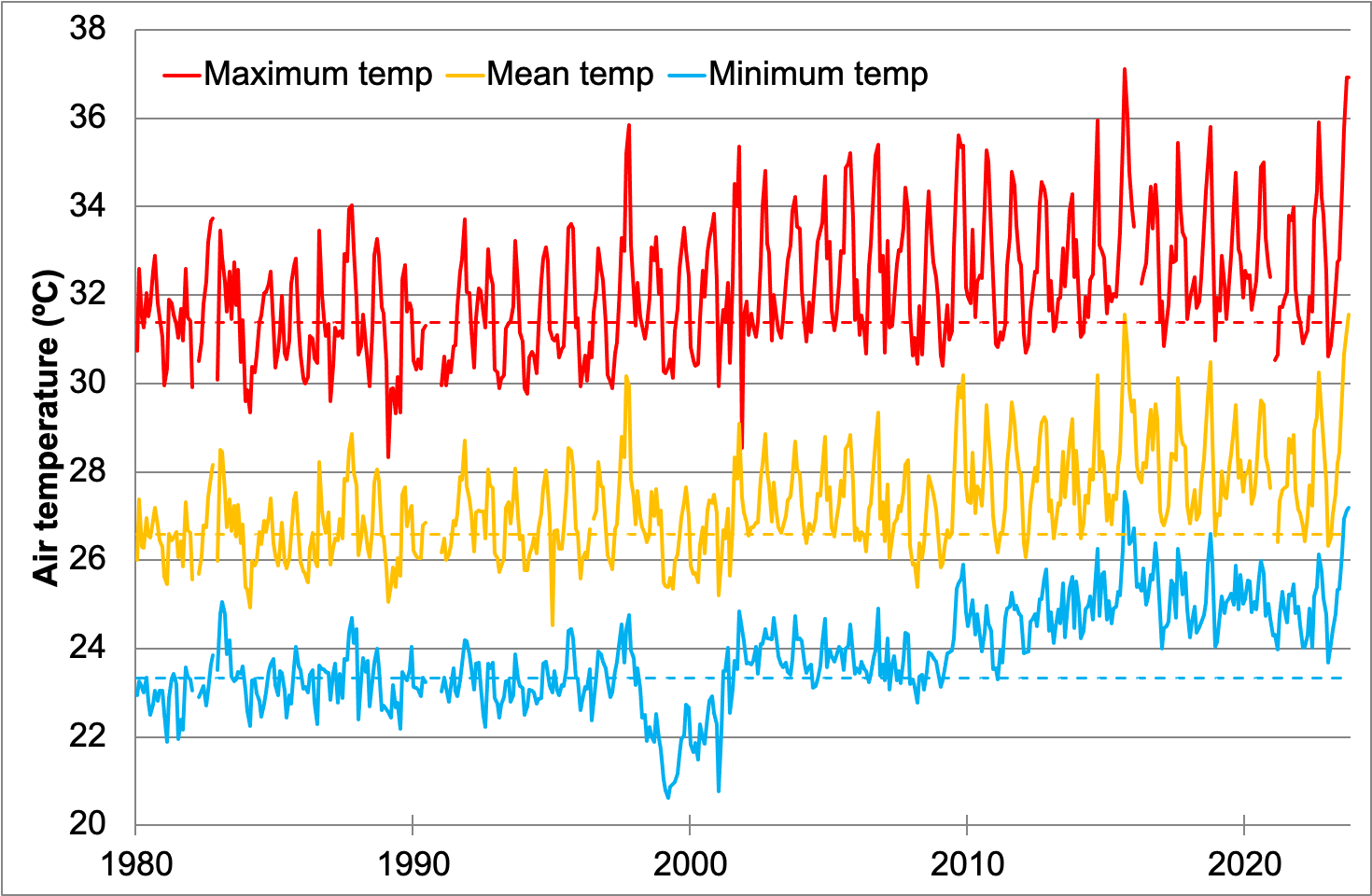


**(a)**


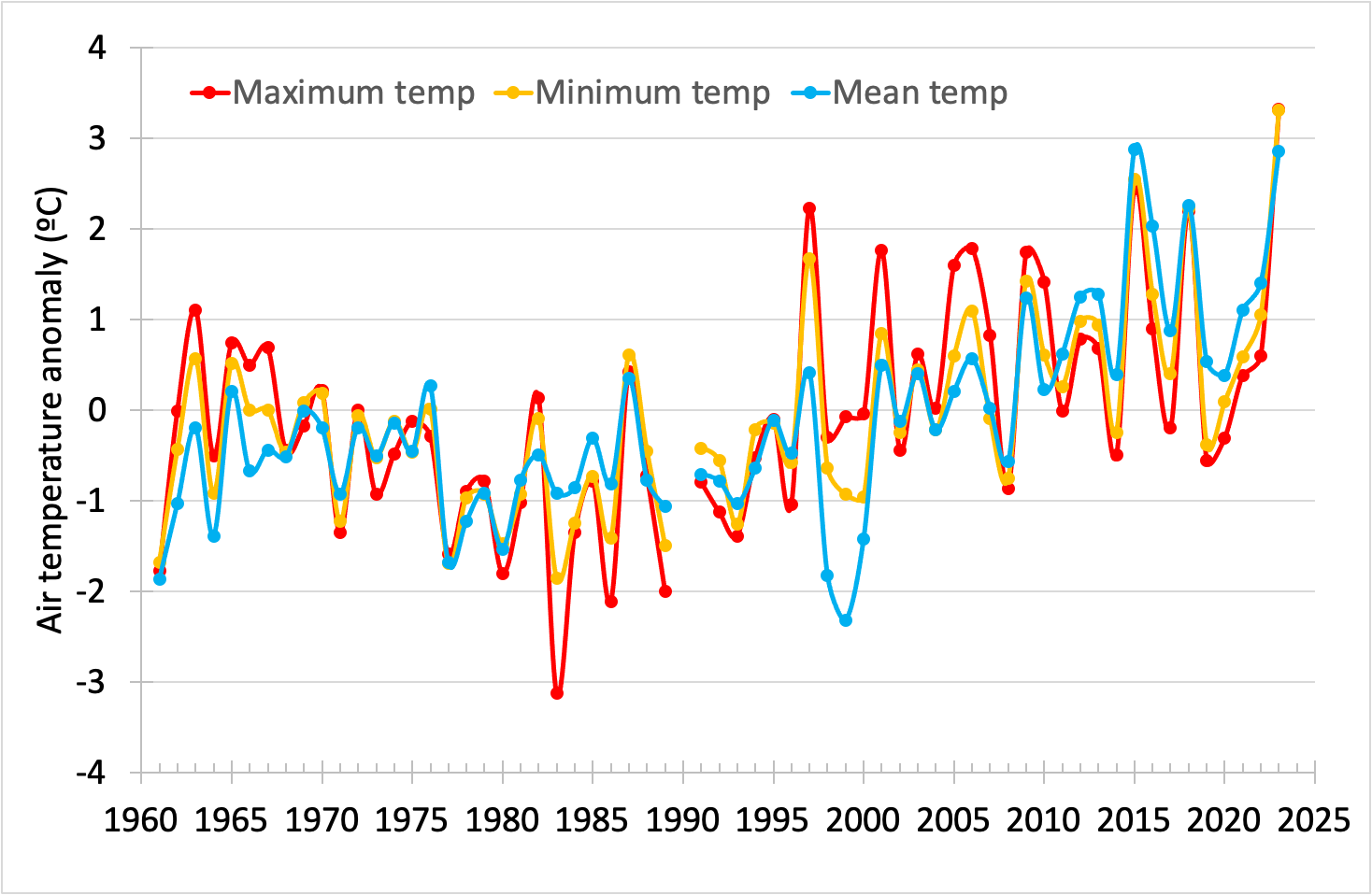


**(b)**

**Supplementary Figure 3.** Monthly maximum, mean and minimum air temperatures registered at the meteorological station of Manaus. (a) Monthly values of air temperature; (b) monthly air temperature anomalies for October.
